# Evidence for Proline Utilization by Oral Bacterial Biofilms Grown in Saliva

**DOI:** 10.3389/fmicb.2020.619968

**Published:** 2021-01-20

**Authors:** Leanne M. Cleaver, Rebecca V. Moazzez, Guy H. Carpenter

**Affiliations:** ^1^Centre for Host Microbiome Interactions, King's College London Faculty of Dentistry, Oral and Craniofacial Sciences, London, United Kingdom; ^2^Centre for Oral, Clinical and Translational Science, King's College London Faculty of Dentistry, Oral and Craniofacial Sciences, London, United Kingdom

**Keywords:** biofilms, proteolysis, metabolomics, NMR spectroscopy, carbon 13 labeling

## Abstract

Within the mouth bacteria are starved of saccharides as their main nutrient source between meals and it is unclear what drives their metabolism. Previously oral *in vitro* biofilms grown in saliva have shown proteolytic degradation of salivary proteins and increased extracellular proline. Although arginine and glucose have been shown before to have an effect on oral biofilm growth and activity, there is limited evidence for proline. Nuclear magnetic resonance (NMR) spectroscopy was used to identify extracellular metabolites produced by bacteria in oral biofilms grown on hydroxyapatite discs. Biofilms were inoculated with stimulated whole mouth saliva and then grown for 7 days using sterilized stimulated whole mouth saliva supplemented with proline, arginine or glucose as a growth-medium. Overall proline had a beneficial effect on biofilm growth—with significantly fewer dead bacteria present by biomass and surface area of the biofilms (*p* < 0.05). Where arginine and glucose significantly increased and decreased pH, respectively, the pH of proline supplemented biofilms remained neutral at pH 7.3–7.5. SDS-polyacrylamide gel electrophoresis of the spent saliva from proline and arginine supplemented biofilms showed inhibition of salivary protein degradation of immature biofilms. NMR analysis of the spent saliva revealed that proline supplemented biofilms were metabolically similar to unsupplemented biofilms, but these biofilms actively metabolized proline to 5-aminopentanoate, butyrate and propionate, and actively utilized glycine. This study shows that in a nutrient limited environment, proline has a beneficial effect on *in vitro* oral biofilms grown from a saliva inoculum.

## Introduction

The oral cavity is a diverse environment where bacteria thrive in complex sessile communities called biofilms. Biofilms in the mouth are exposed to intermittent variations of temperature through food and fluid consumption, osmolality, anti-microbials, pH, and nutrient availability. Oral bacteria within biofilms are symbiotically reliant on each other for increased survival or virulence in disease. Studies have shown that when compared to mono-culture, some oral bacteria grown in co-culture exhibit greater growth and changes in global gene expression leading to activation of different metabolic pathways (Tan et al., 2014). Oral health has been described as a balance between proteolytic and glycolytic pathways (Zaura et al., 2017). Too much glycolytic activity leads to lactic acid production and caries whereas too much proteolytic activity is associated with periodontitis. Yet both activities are a normal part of the oral microbiome, with glycolytic activity dominant during the consumption of foods and drinks and proteolytic activity being dominant in between. If oral health is a balance between proteolytic and glycolytic activity then understanding the factors that affect this balance may identify strategies to treat dysbiotic states.

Metabolomics of oral biofilms—the large-scale study of molecules that derive from the metabolism or catabolism of organic compounds—is being utilized more frequently to understand how bacteria are interacting when grown in complex communities, both *in vivo* (Gardner et al., 2019) and *in vitro* (Takahashi et al., 2010). Metabolomics has been used to define oral health and disease using methodologies such as capillary electrophoresis (CE) paired with time-of-flight mass spectrometry (TOFMS) (Sugimoto et al., 2010) and liquid chromatography tandem mass spectrometry (LC-MS/MS) (Ghannoum et al., 2013).

In our previous paper, NMR was used to assess the metabolic activity of *in vitro* oral biofilms inoculated using stimulated whole mouth saliva, and grown using sterilized stimulated whole mouth saliva as a growth-medium (Cleaver et al., 2019). Using SDS-polyacrylamide gel electrophoresis (SDS-PAGE) we were able to show that in this nutrient-depleted growth-medium, proteolysis of salivary proteins correlated with biofilm growth. As proteolytic activity of the biofilms increased, there were significantly increased levels of the amino acids and in particular, proline. Since proline-rich proteins (PRPs) are the most abundant proteins in saliva and the most degraded group of salivary proteins, we therefore hypothesized that PRPs are the source of proline and that this amino acid can be utilized by bacteria in biofilms. In the present paper we go on to assess whether proline can be utilized by oral bacteria and indeed if it should be considered a prebiotic.

Prebiotic is a term that is used to describe a compound or molecule that can alter bacterial growth or metabolic activity, which then provides beneficial effects to the host (Roberfroid, 2007). This was updated in 2017 to include any substrate that can be selectively utilized by microorganisms (Gibson et al., 2017). This is different to probiotics, which is the ingestion of bacteria with the aim to promote a beneficial health consequence. In the literature prebiotics have previously been described in the gut microbiota and were defined as non-digestible oligosaccharides (Davani-Davari et al., 2019)—so as they reach the gut unaltered.

The interest in prebiotics for microbiome alteration has unsurprisingly become a popular research topic in the field of oral microbiology. This is likely due to evidence which suggests that the oral microbiome cannot be easily altered by introducing non-resident bacteria into the oral cavity (Kilian et al., 2006). Oral prebiotics work by providing resident non-pathogenic bacterial species with a carbon or nitrogen source, with the aim to outcompete pathogenic bacteria, which results in a “healthier” oral cavity. Different substrates have been used so far; Tester and Al-ghazzewi (2008) used konjac glucomannan hydrosylate to significantly increase the growth of *Lactobacillus acidophilus* (a non-pathogenic bacteria) compared to *Streptococcus mutans* (a pathogenic bacteria), and (Kojima et al., 2016) have used a prebiotic (arabinose, xylose and xylitol) and probiotic (*Lactobacillus* strains) concurrently to inhibit the growth of pathogenic species *Candida albicans* and *Porphyromonas gingivalis*.

Glucose is rapidly broken down by oral bacteria, particularly *S. mutans* and certain *Lactobacillus species*, into acids which cause a rapid drop in pH (Muntz, 1943) which can lead to caries and the dissolution of exposed enamel (Loesche, 1986; Bowden, 1990; Liljemark and Bloomquist, 1996; Marsh, 1999). Numerous studies have shown that the production of acids by oral bacteria that metabolize glucose and other dietary carbohydrates leads to dysbiosis of plaque bacteria; acid tolerant bacterial species proliferate, and acid sensitive bacterial species decrease. The greater the acid secretion, the more tooth enamel dissolves. Therefore, glucose can alter bacterial growth and metabolism, but does not confer a benefit to the host, so cannot be described as a prebiotic.

In contrast, arginine, a semi-essential amino acid, is widely regarded as a prebiotic in the literature. It has been shown to counteract the consequences of glucose metabolism, and produces a beneficial effect to the host (reduction in caries; Huang et al., 2016). Arginine can be broken down by certain bacteria in oral biofilms into putrescine and ammonia via the agmatine deiminase and arginine deiminase pathway (Nascimento et al., 2019). The ammonia produced increases the pH in the mouth, allowing a shift in microbiota to a more diverse non-pathogenic community (Agnello et al., 2017). The increase in pH due to arginine consumption by bacteria also favors enamel remineralisation and decreases mineral loss, which has a positive effect on hard tissues in the mouth (Yamashita et al., 2013; Yu et al., 2016; Bijle et al., 2018).

Proline has recently been shown to have a role in pathogen and host interactions outside of the oral cavity in many different bacterial species - including *Escherichia coli, Staphylococcus aureus*, and *Mycobacterium tuberculosis*- by modulating osmotic stress, cell signaling, and in some cases by breaking down proline into metabolites that have antibacterial properties (Christgen and Becker, 2019). There are a limited number of studies that investigate the metabolism of proline by oral bacteria in reference to its use as a prebiotic. One study that used a proline-containing pentapeptide, RGRPQ, which was identified as a breakdown product of PRP-1 by oral bacteria, showed that the peptide was able to affect biofilm adhesion and proliferation by *Streptococcus gordonii* (a beneficial commensal) and resist sucrose-induced pH decrease (Drobni et al., 2006). Slomka et al. successfully presented data in support of a proline-containing prebiotic candidate, methionine-proline, that shifted the composition of a 14-species oral biofilm model by reducing pathogenic species to a predominantly beneficial species population after just 3 days exposure to the prebiotic (Slomka et al., 2017, 2018).

Based on our previous work which suggests that bacteria degrade PRPs and produce increased levels of proline, the aim of this study was to assess the ability of proline to affect the growth, proteolytic activity and metabolic activity of supplemented healthy *in vitro* oral biofilms compared to known mediators of biofilm growth and activity, arginine and glucose.

## Materials and Methods

### Saliva Collection

Fourteen informed participants, who had given consent, produced a stimulated whole mouth saliva sample by chewing on unflavoured paraffin film and expectorating for ~10–15 min until 10 ml saliva was collected. Samples were provided in the afternoon, at least 1 h after eating and drinking, apart from water. Volunteers were not required to refrain from oral hygiene practices prior to participation. Two samples were collected on separate days. Participants were included in the study if they self-reported as systemically and orally healthy and had not taken antibiotics in the preceding 3 months. Participants were a mix of male and female aged over 18 years. This study was approved by the King's College London Research and Ethics Committee, reference HR-17/18-6116.

### Saliva Processing and Inoculation and Incubation of Hydroxyapatite Discs

Saliva was processed and biofilms were grown using a previously published methodology with modifications (Cleaver et al., 2019). Saliva samples were centrifuged (5,000 × g for 5 min), and the supernatant was collected, pooled, boiled in sealed tubes for 20 min to sterilize, and left to cool to room temperature; hereafter referred to as sterile saliva. Unpublished data by our group has previously shown that boiling saliva does not change the composition of salivary proteins present, but does reduce the amount of amylase present in line with other studies (Pramanik et al., 2001), and is preferable over filtering as the small pore size for filtering is easily blocked by saliva. The loose pellet from unboiled, spun saliva samples was pooled and combined with enough sterile saliva to facilitate inoculation of the hydroxyapatite (HA) discs. Samples were vortexed vigorously to resuspend the pellet; this constituted the pooled saliva inoculum and was used on the same day as processing.

The remaining sterile saliva was split into 12 aliquots. L-proline, L-arginine, and glucose were added to the aliquots to achieve concentrations of 10, 25, and 50 mM, respectively, for each [based on concentrations of proline obtained from previous publication (Cleaver et al., 2019)]. One aliquot was supplemented with 10 mM carbon 13 labeled (C^13^) L-proline. Two aliquots were left unsupplemented to act as a positive and negative control. Aliquots were frozen, then gently thawed overnight at 4°C before use.

Six HA discs in a sterile microtitre plate were inoculated per treatment condition with 1 ml pooled saliva inoculum (negative control discs were incubated with sterile saliva only, to check for sterility) and incubated for 24 h aerobically in a 40 L aerobic incubator (GenLab Ltd., UK) at 37°C. After this time the discs were washed three times with sterile phosphate buffered saline (PBS, 137 mM NaCl, 2.7 mM KCl, and 10 mM phosphate buffer, pH 7.4) and 1 ml of supplemented/unsupplemented saliva per condition was added to the discs. The discs were then incubated anaerobically in a 3.5 liter anaerobic jar with an Anaerogen anaerobic generator pack (Oxoid, UK) at 37°C for 72 h. After this spent saliva from the biofilms was removed and stored at −20°C for further analysis, discs were washed three times with PBS, and refreshed with supplemented/unsupplemented saliva and further incubated anaerobically at 37°C for 72 h. After this final incubation the spent saliva was removed and stored at −20°C for further analysis. The mixed initial aerobic then anaerobic incubation of discs has been chosen based on our previous findings, that showed mixed incubation environments induced proteolysis, and the findings of co-aggregation studies, which suggest that aerobic bacteria are initial colonizers that subsequently facilitate the binding of anaerobic bacteria into the biofilm (Diaz et al., 2006; Periasamy and Kolenbrander, 2010; Cleaver et al., 2019).

### Analysis of Biofilm Growth

At day 7, three HA discs per treatment were washed three times with sterile PBS after which 50 μl of both SYTO-9 (final concentration 6 μM) and propidium iodide (PI; final concentration 30 μM) resuspended in sterile PBS (LIVE/DEAD™ BacLight™ Bacterial Viability Kit, ThermoFisher Scientific, USA) were added to each disc. Discs were incubated for 15 min at room temperature prior to analysis with a DM-IRE2 confocal laser scanning microscope (Leica Microsystems Heidelberg GmbH, Germany).

Five random z-series stacks (z-height divided into 10 images, from each quadrant and the middle of each disc) were captured at x63 magnification using the Leica Microsystems Confocal Software (version 2.61 Build 1537). Images were analyzed using ImageJ Software (version 1.52) using the Comstat2 plugin [version 2.1 (Heydorn et al., 2000; Vorregaard, 2008)]. Biomass (μm^3^/μm^2^) was captured and the average results of all 5 z-series stacks was calculated for each measurement. Fluorophore beads of known size were used to calibrate z-height.

### Measurement of pH

Spent saliva samples from all biofilms including the unsupplemented control were measured for pH using a micro pH probe (Jenway, Cole-Palmer Scientific, Hong Kong). The average and standard deviation of three replicates was calculated per un/supplemented biofilm, including the sterile saliva growth-medium.

### Protein Analysis by SDS-Polyacrylamide Gel Electrophoresis (SDS-PAGE)

Three spent saliva samples from each disc per treatment were centrifuged at 16,000 × g for 5 min. A 15 μl aliquot of supernatant was added to 5 μl of loading dye and 1 μl of dithiothreitol, and 15 μl of this was loaded onto a ready-made 4–12% Bis-Tris polyacrylamide gel (Invitrogen, USA), run for 32 min at 200 volts constant, stained with Coomassie brilliant blue R solution (Merck, Germany) and de-stained with 10% acetic acid. A ChemiDoc (Bio-Rad, USA) was used to capture gel images, which were analyzed using Image Lab (Bio-Rad) using protein volume intensity tools (whole lane and protein band).

### Nuclear Magnetic Resonance (NMR) Metabolic Analysis

Samples were processed for NMR using a previously published method (Cleaver et al., 2019). Briefly, centrifuged spent saliva supernatant and the sterile saliva sample were each mixed with TSP buffer (one part 2 mM sodium trimethylsilyl-[2,2,3,3-^2^H_4_]-propionate, 28.4 mg/ml Na_2_HPO_4_ and 5.28 mg/ml NaH_2_PO_4_ to two parts 50% by volume deuterium oxide, sample:buffer ratio 4:1) in a 5 mm NMR tube (Bruker, Germany). The tubes were sealed and analyzed at the Biomolecular Spectroscopy Centre, King's College London, UK on a 600 MHz spectrometer (Bruker) for ^1^H 1D-NMR and ^1^H-C^13^ 1D- and 2D-NMR. The concentration of metabolites were collected using Chenomix NMR Suite version 8.5 (Chenomix Ltd., Canada). The 2D C^13^ spectra were analyzed using TopSpin version 3.6.2 (Bruker) and COLMAR (Bingol et al., 2014).

### Statistical Analysis

All data were analyzed for normality using the Shapiro-Wilks normality test. All analyses were performed using one-way ANOVA if they passed normality or Kruskal-Wallis if they did not pass normality, then were compared to the unsupplemented control by multiple comparison using GraphPad Prism (version 8). Principal component analysis was performed using the libraries ggplot2, ggfortify, stringr, screeplot and cluster in RStudio (version 3.6.1).

## Results

### Biofilm Growth and pH Measurement

#### Biomass

**Figure 1:**
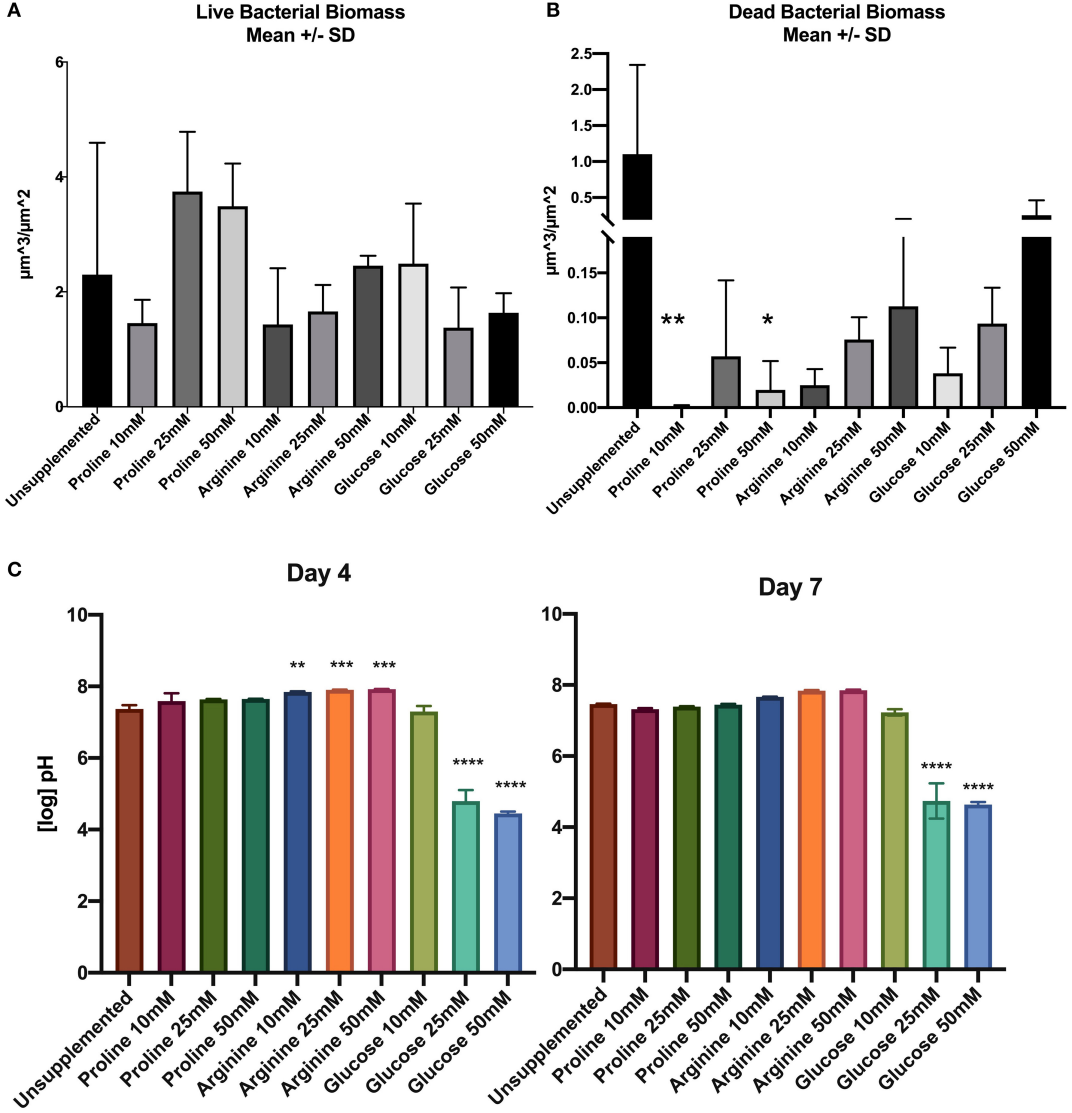
**(A)** Live bacterial biomass, **(B)** dead bacterial biomass, **(C)** pH of spent saliva growth medium. Units for biomass are μm^3^/μm^2^ (*n* = 3; ^*^p < 0.05, ^**^p < 0.005, ^***^p < 0.001, ^****^p < 0.0001).

When compared to the positive control, there was no significant difference in live bacterial biomass between any of the treated biofilms and the control. Although there were no significant differences for live bacterial biomass, proline at 25 and 50 mM concentrations had a greater average biomass than the control, and as the arginine concentration increased the average live biofilm biomass also increased (Figure 1A).

The dead bacterial biomass data did not pass Shapiro-Wilks normality testing; therefore Kruksal-Wallis multiple comparisons were performed. The amount of dead bacterial biomass of all treated biofilms was lower than the live bacterial biomass (Figure 1B). When the concentration of arginine and glucose increased, the dead bacterial biomass also increased.

Unsupplemented biofilms had high proportions of dead bacterial biomass compared to all of the treated biofilms. Supplementation of biofilms with proline at 10 and 50 mM appears to have a significant effect on the biofilm by decreasing the proportion of dead bacteria in the biomass.

#### Measurement of pH

Figure 1C shows the pH of the starting saliva and the days 4 and 7 biofilm spent saliva (totalling three separate pH measurements; days 0, 4, and 7). Growth medium was refreshed at days 1 and 4, therefore the pH measurement was not continuous over the 7 days. The unsupplemented biofilm and all concentrations of proline supplemented biofilm samples remain at a neutral pH for all time points. All concentrations of arginine starting saliva were between pH 8.8–9.3, which decreased at day 4, but was still significantly higher than the unsupplemented control, and decreased further to pH 7.6–7.7 on day 7. The pH of glucose 10 mM was not significantly different to the unsupplemented control at days 4 and 7. However, biofilms supplemented with 25 and 50 mM glucose were significantly lower than the unsupplemented control at day 4 (pH 4.5–4.6) and at day 7 (pH 4.5–4.7).

### Protein Analysis by SDS-PAGE

All spent saliva from unsupplemented and supplemented biofilms was assessed for salivary protein degradation by comparing the separated protein profile (total lane volume and presence of protein bands) of the sterile saliva with that of the spent saliva. The negative control (sterile saliva incubated on HA disc) demonstrated no degradation of salivary proteins, suggesting limited endogenous protease activity (see Supplementary Figure 2). The unsupplemented control biofilm demonstrated protein degradation, despite the lack of supplementation, which suggests that bacteria in biofilms that are grown in nutrient limited media will degrade proteins.

**Figure 2:**
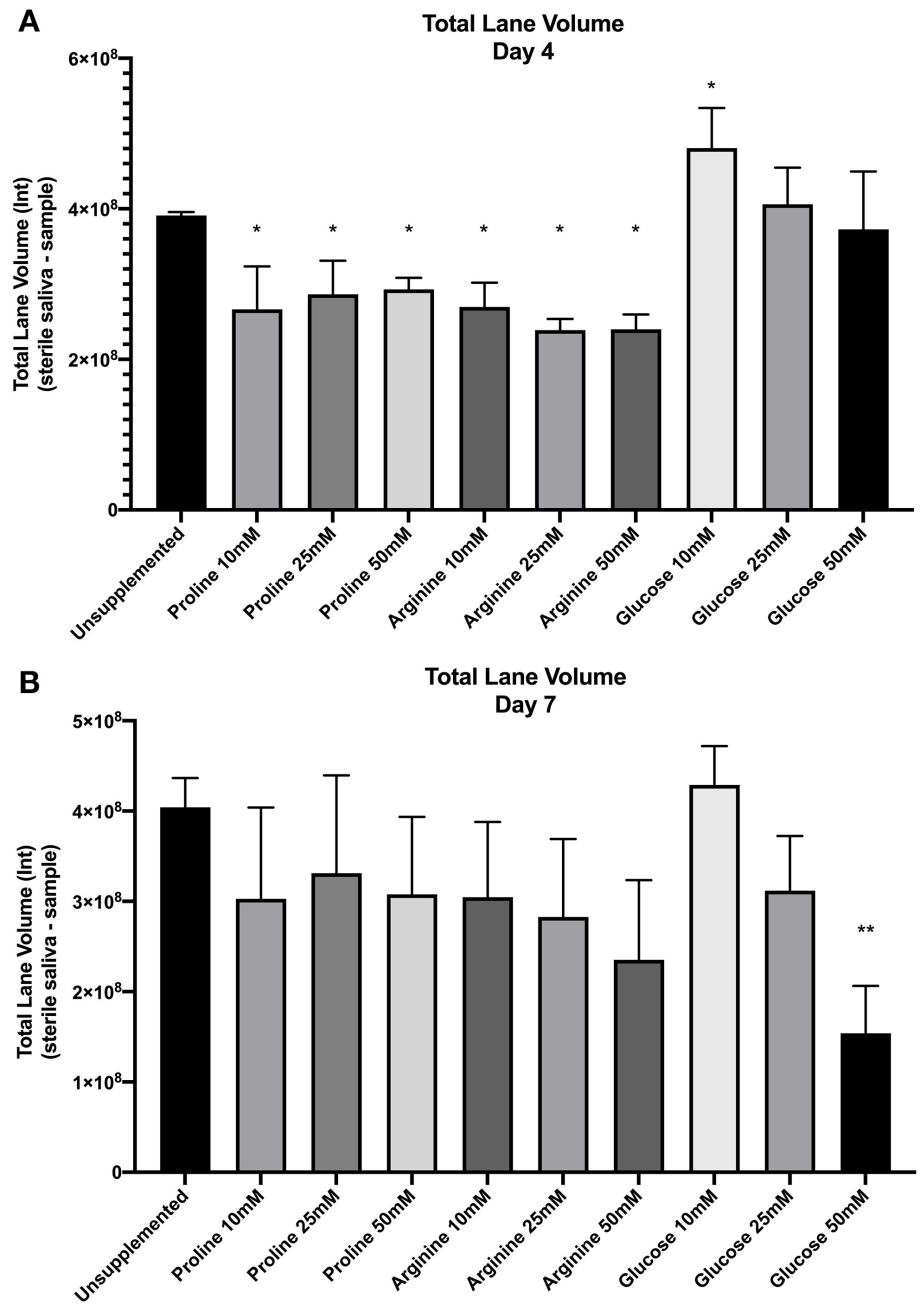
SDS-PAGE total lane volume (arbitrary intensity units; Int) of biofilm spent saliva at day 4 **(A)** and day 7 **(B)**. Total lane volume was calculated as the total lane volume intensity of the sterile saliva growth medium minus the total lane volume intensity of the spent saliva sample (*n* = 3; ^*^p < 0.05, ^**^p < 0.005).

Figure 2 shows the total lane volume of both unsupplemented and supplemented biofilms (mean ± SD, *n* = 3). The total lane volume for spent saliva from biofilms was subtracted from the total lane volume of sterile saliva to normalize data between gels to calculate the loss of protein. A smaller value equates to less protein degradation.

Total lane volume of all supplemented biofilm spent saliva samples was compared by one-way ANOVA and multiple comparison with Benjamini, Krieger and Yekutiele false discovery rate against the unsupplemented control to determine whether proline, arginine or glucose inhibited or promoted further salivary protein degradation. At day 4 all concentrations of proline and all concentrations of arginine had significantly lower total lane volume (*p* < 0.05) than the unsupplemented control, therefore there was less protein degradation by these biofilms. Spent saliva from biofilms grown in the presence of glucose at 10 mM had a significantly higher total lane volume (*p* < 0.005) at day 4, suggesting more protein degradation in these biofilms. At day 7, only the spent saliva from glucose 50 mM supplemented biofilms had a significantly lower total lane volume compared to the unsupplemented control, suggesting that at day 7 there was less salivary protein degradation by the bacteria in these biofilms. Therefore, proline and arginine at all concentrations inhibited protein degradation and glucose 10 mM induced protein degradation in less mature biofilms whereas glucose 50 mM inhibited protein degradation in more mature biofilms.

The volume (intensity) of distinct protein bands in the gels was determined. The most distinct proteins were identified based on molecular weight. The intensity of these bands was divided by the intensity of the corresponding band in the sterile starting saliva to give a ratio that could be compared across gels – a lower ratio equated to increased protein degradation. The identified proteins were; mucin 5b, mucin 7, glycosylated PRP, amylase, acidic PRP, basic PRP, cystatin, statherin, and histatin. Shapiro-Wilks normality was conducted on each band for days 4 and 7. If the results passed normality testing then one-way ANOVA with multiple comparisons was performed, if not then Kruskal-Wallis with multiple comparisons was performed. Statistical analysis showed no significant difference between the unsupplemented control proteins and supplemented biofilm proteins at day 4, except glucose 10 and 25 mM significantly reduced the volume of mucin 7 (*p* < 0.05 and *p* < 0.05, respectively) and arginine 25 and 50 mM significantly increased the volume of basic PRP (*p* < 0.05 and *p* < 0.001, respectively). At day 7, none of the protein bands were significantly different when compared to the unsupplemented control, except glucose 10 mM significantly decreased the volume of amylase (*p* < 0.05). The lack of statistical significance may have been due to inter-gel variation. Therefore, data from all gels was normalized against the sterile saliva and expressed as a ratio. However, the results remained the same. These results suggest that for biofilms supplemented with glucose 10 mM, the reduction of mucin 7 (day 4) and amylase (day 7) may be contributing to the overall increased loss of total volume of salivary proteins in these biofilms. The results also suggest that biofilms supplemented with arginine 25 and 50 mM may inhibit the degradation of basic PRPs which may be contributing to the overall decrease in total volume of salivary proteins in these biofilms.

### NMR Analysis

#### ^1^H 1D Spectral Analysis

All 1D ^1^H spectra were uploaded into Chenomx and peaks were automatically assigned and fitted to give the concentration of individual metabolites. These were then normalized against the metabolites in the negative control, to control for changes to metabolite concentration as a result of incubation of the saliva (a negative value indicates the metabolite that was present in the sterile saliva growth medium has been used up by the bacteria in the biofilms). Nineteen metabolites were identified overall in the samples, including short chain fatty acids, organic acids, and amino acids. Statistical analysis was performed on the 8 most distinguishing metabolites between treatments; putrescine, butyric acid, 5-aminopentanoate, lactate, acetic acid, propionate, glycine and proline. Shapiro-Wilks normality testing was performed on the data for each metabolite and if normally distributed one-way ANOVA with multiple comparison to the unsupplemented control was performed and if not normally distributed Kruskal-Wallis with multiple comparison to the unsupplemented control was performed.

**Figure 3:**
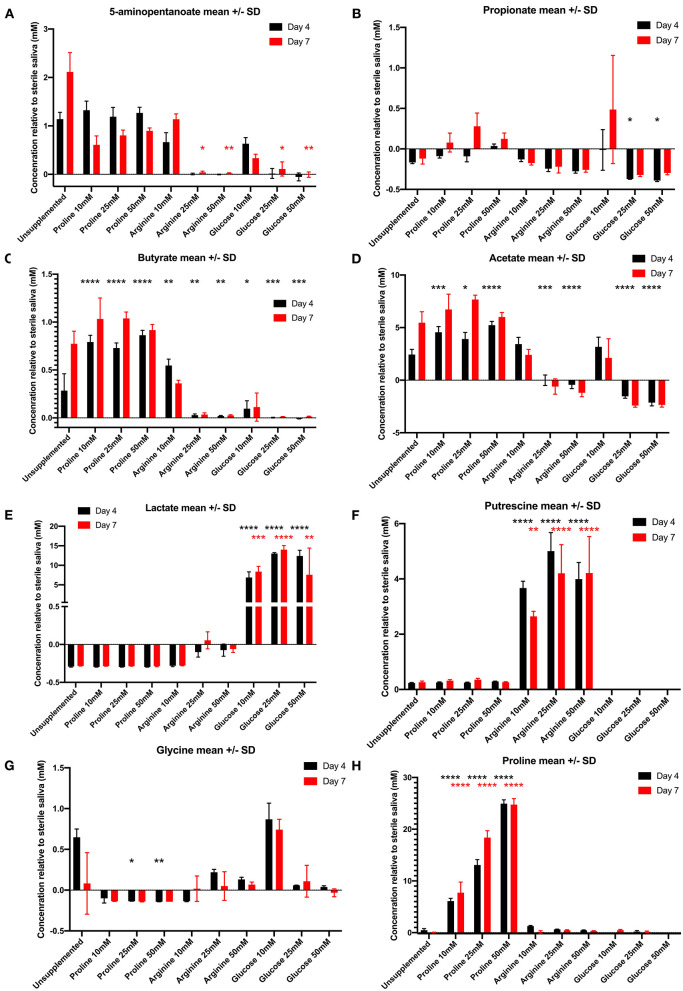
Concentration of metabolites (mean ± standard deviation) in day 4 (black bars) and day 7 (red bars) in unsupplemented and supplemented biofilms relative to the sterile saliva media. Negative concentrations (less than zero) indicate the metabolite has been consumed from the sterile saliva growth medium by bacteria in biofilms. **(A)** 5-aminopentanoate, **(B)** propionate, **(C)** butyrate, **(D)** acetate, **(E)** lactate, **(F)** putrescine, **(G)** glycine, **(H)** proline (*n* = 3; ^*^p < 0.05, ^**^p < 0.005, ^***^p < 0.0005, ^****^p < 0.0001).

Figure 3A shows the concentrations of 5-aminopentanoate, also known as 5-aminovalerate, produced by unsupplemented and supplemented biofilms. Biofilms supplemented with 25 and 50 mM arginine and glucose had significantly lower levels of this metabolite at day 7 than the unsupplemented control. There was no significant difference for proline supplemented biofilms at either timepoint. Propionate (Figure 3B) displayed varying concentrations across all supplemented biofilms. Although not significantly different, at day 7 all proline concentrations resulted in higher concentrations than the unsupplemented control. This suggests it is one of the metabolites downstream from proline. Another metabolite that could possibly be a product of proline degradation is butyrate (Figure 3C). At day 4 all proline supplemented biofilms had significantly higher concentrations of butyrate compared to the unsupplemented control—all other supplemented biofilms (except arginine 10 mM) are significantly lower than the unsupplemented control. Biofilms supplemented with proline also produced significantly more acetate than the unsupplemented control and the other supplemented biofilms at day 4 (Figure 3D). Lactate (Figure 3E) was produced in significantly higher concentrations for all glucose supplemented biofilm concentrations at both timepoints, in contrast to this metabolite being used up by proline exposed biofilms and the unsupplemented control. This might explain the extreme acidity of glucose supplemented biofilms. Putrescine (Figure 3F) was the most significant result for arginine supplemented biofilms, which produced significantly greater concentrations than all other biofilms—likely via the agmatine pathway. Glucose supplemented biofilms produced no putrescine at all. The concentration of glycine (Figure 3G) is significantly lower than the unsupplemented control for biofilms supplemented with proline 25 and 50 mM—in fact, it is being used up by all proline concentrations and arginine 10 mM. As expected proline was detected in high levels in the proline-supplemented biofilms (Figure 3H) but at only half the concentrations added; proline 10 mM drops to a mean concentration of 6 mM on day 4 and 7 mM on day 7, proline 25 mM drops to a mean concentration of 12 mM on day 4 and 18 mM on day 7, and proline 50 mM drops to a mean concentration of 25 mM on both days. These results suggest that these two amino acids, glycine and proline, are being consumed and degraded by oral bacterial biofilms *in vitro*.

**Figure 4:**
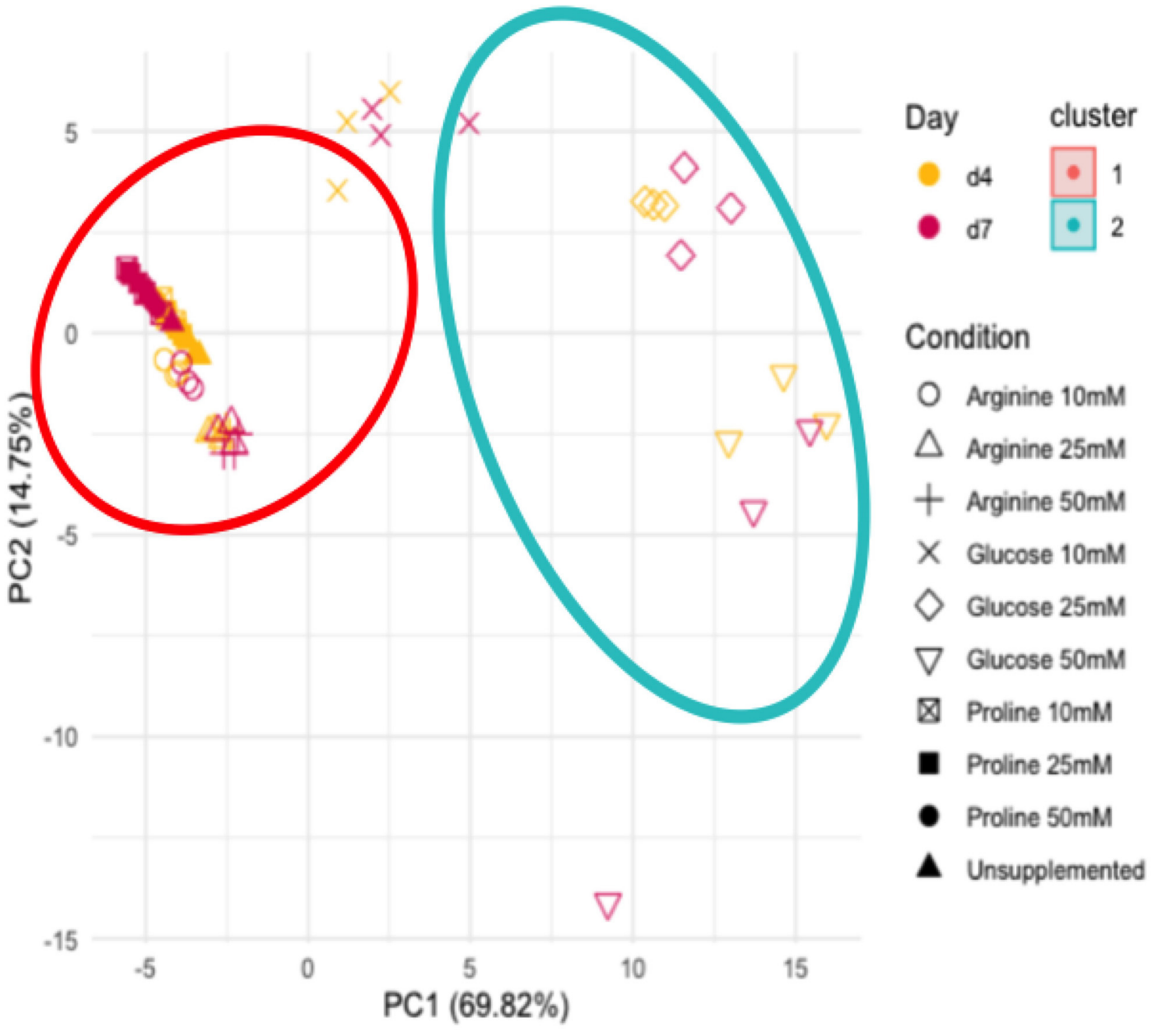
Principle component analysis of all metabolite concentrations, unsupplemented and supplemented biofilms and the sterile saliva. Unsupplemented, unsupplemented control.

To determine whether the metabolites detected in the supplemented biofilm samples were similar or varied in their metabolic profile, principle component analysis (PCA) was performed (Figure 4). PCA was performed on data normalized to the negative control for all 19 metabolites with measured proline concentrations removed. The PCA shows that there are two distinct clusters of samples, as determined by the screeplot and kmeans cluster libraries within RStudio. The unsupplemented control and all concentrations/time points of arginine and proline cluster tightly together (cluster 1), suggesting that the metabolic profile of arginine and proline supplemented biofilms is not dissimilar from the biofilms that have no supplementation. The samples from biofilms supplemented with glucose cluster closely (cluster 2), with the 10 mM concentration clustering further toward the unsupplemented control along the x axis (PC1). Therefore, biofilms supplemented with amino acids do not dominate metabolism in the same way that glucose does.

### 1D ^1^H and 2D ^1^H-C^13^-Labeled Proline Spectral Analysis

**Figure 5:**
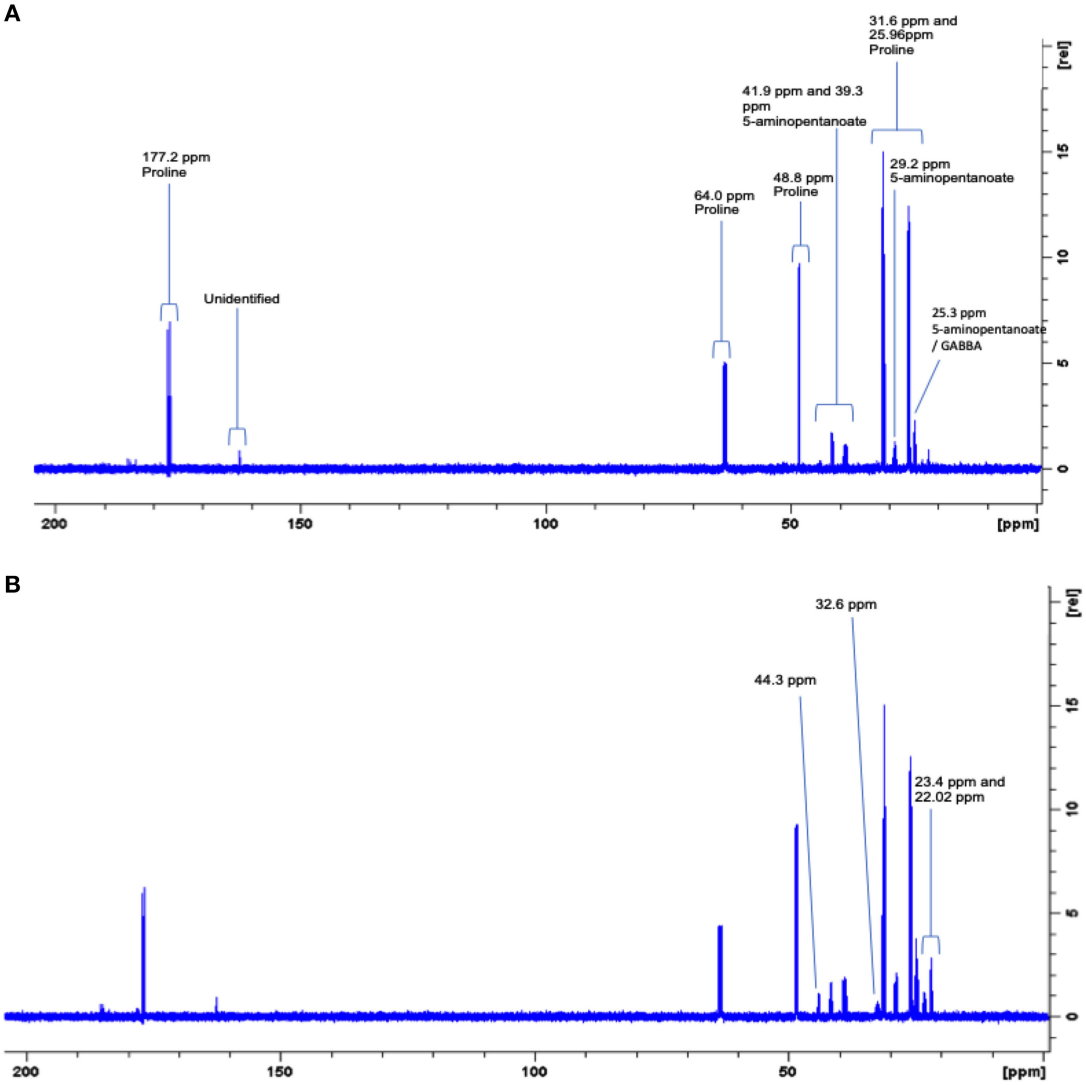
Representative full 600 MHz 1D C^13^ NMR spectra for one day 4 **(A)** and day 7 **(B)** 10 mM C^13^-labeled proline supplemented biofilm spent saliva. Identification and chemical shift (ppm) of metabolite peaks are labeled in **(A)** and additional peaks identified at day 7 are labeled in **(B)**.

**Figure 6:**
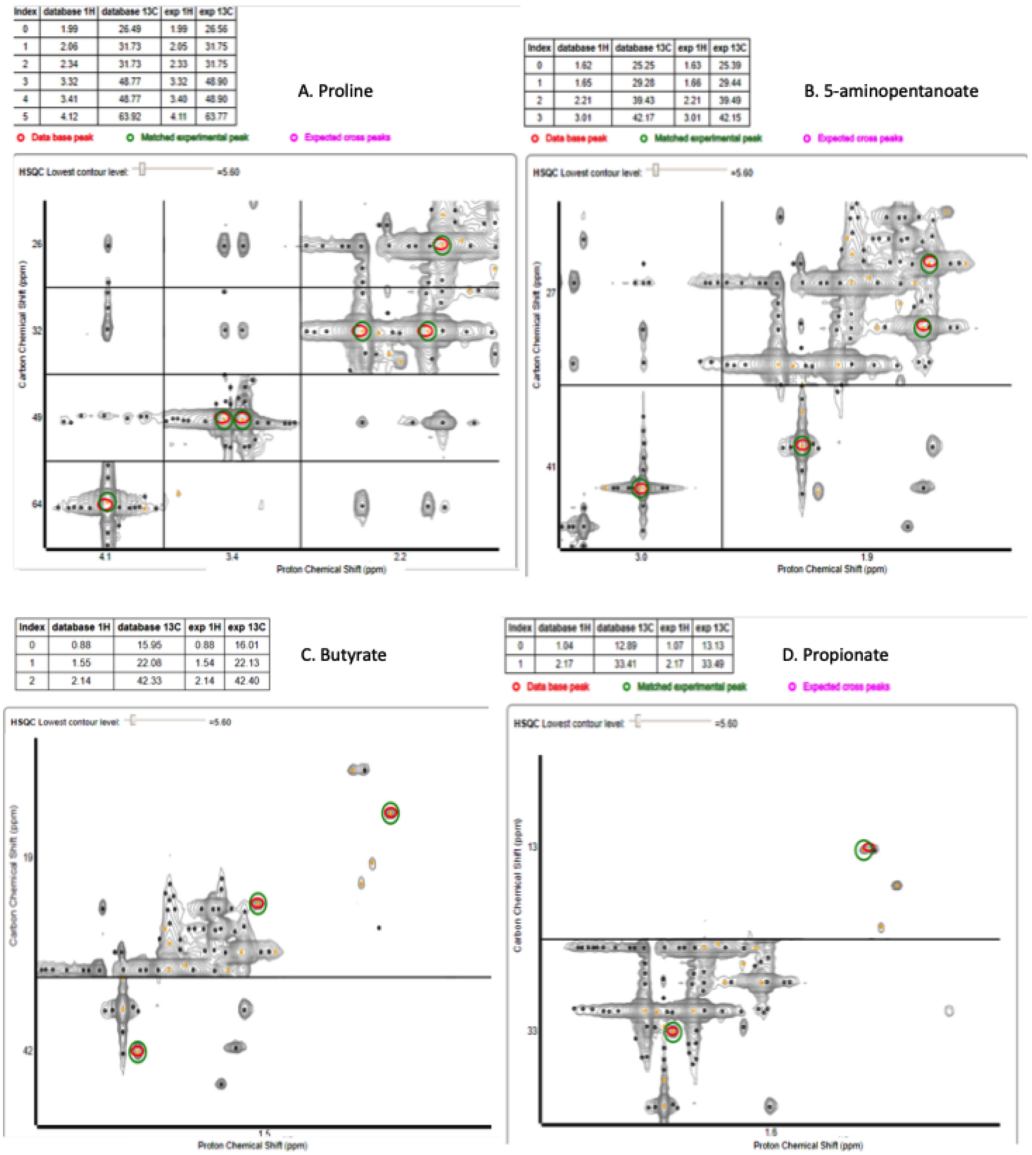
2D HSCQ NMR spectra for one day 7 10 mM C^13^-labeled proline supplemented biofilm spent saliva sample. The red circles identify the peaks within the database, and the green circles identify the matched experimental peak from the sample. The experimental peaks match all database peaks for proline **(A)**, 5-aminopentanoate **(B)**, butyrate **(C)**, and propionate **(D)**.

Since these biofilms appear to use proline, they were supplemented with C^13^-labeled proline to identify the products and possible pathways of proline breakdown. The 1D spectra produced by spent saliva from biofilms that were supplemented with C^13^-labeled proline (10 mM) were analyzed by identifying peak shifts in TopSpin and correlating these to the Human Metabolome Database, and one day 7 biofilm replicate subjected to 2D NMR was analyzed using a ^1^H–^13^C heteronuclear single quantum correlation (HSQC) untargeted metabolome database (Bingol et al., 2014). As can be seen in Figure 5A, the 1D spectral peaks for day 4 C^13^-proline supplemented biofilm spent saliva are identified as proline (25.96, 31.6, 48.8, 64.0, and 177.2 ppm) and 5-aminopentanoate/gamma aminobutyric acid (25.3, 29.2, 39.3, and 41.9 ppm). There is also a peak at 162.8 ppm that does not fit with any salivary metabolites and at present is unidentifiable. Figure 5B shows the 1D spectral peaks for the same replicate for day 7 C^13^-proline supplemented biofilm spent saliva, which also shows the proline and 5-aminopentanoate peaks but also 3 additional peaks at 22.02, 23.4, 32.6, and 44.3 ppm. The 2D HSQC spectra identified proline and 5-aminopentanoate suggesting a Stickland reaction, and additionally butyrate and propionate (Figure 6) which are breakdown products of amino acids.

## Discussion

In this paper we show oral biofilms readily metabolize proline to produce 5- aminopentanoate, butyrate, and propionate, all of which are major metabolites found in saliva (Cleaver et al., 2019). The degradation of proline to 5-aminopentanoate is likely through a Stickland reaction mechanism or proline reductase (Curtis et al., 1983; Kabisch et al., 1999; Bouillaut et al., 2013). The sequential shortening of 5-aminopentanoate would next lead to gamma aminobutyric acid (GABA) which has also been found in saliva (Silwood et al., 2002) and is tentatively assigned here based on 1D C^13^ NMR spectra. Although proline did not increase the biomass of the biofilm it did reduce the amount of visualized dead bacteria in the biofilm. This suggests that the uptake and degradation of this amino acid is sufficient to maintain growth of bacteria when saccharides, such as glucose and sucrose, are not present and that this is a major pathway in the metabolism of oral bacteria. However, when sugars are present, as shown by the glucose supplemented biofilms, the proline utilization pathway is almost completely turned off. Acetate, 5-aminopentanoate and butyrate secretion did not rise above background levels by the addition of glucose (>10 mM). Arginine supplementation had a similar effect and at concentrations over 10 mM it also reduced components of the proline breakdown pathway. Arginine and glucose supplemented biofilms continued to grow and the proportion of dead bacteria within the biofilm biomass was also reduced compared to unsupplemented biofilms. In comparison to glucose, proline appears to be beneficial as it did not increase lactate production and did not decrease the pH of the surrounding medium below neutral. Thus, it might be concluded that the proline breakdown pathway can maintain oral bacteria in low nutrient conditions but is not necessary for growth if other nutrients are added.

In addition, proline supplementation altered the biofilms from glycine-producing to glycine-utilizing, although the potential consequences of this are not clear at present. Proline supplementation of growth medium has been shown in *Clostridium difficile* to upregulate a specific proline reductase gene (pdrR) and glycine reductase genes for preferential utilization of proline and glycine in this organism (Bouillaut et al., 2013), which will be investigated by our group in future experiments. Glycine has previously been shown to have immune modulating effects. Several studies have shown that glycine has an anti-inflammatory role, not just in the oral gingiva (Schaumann et al., 2013), but also in sepsis (Spittler et al., 1999) by reducing tumor necrosis factor alpha (TNF-α). In contrast to these results, others have shown that glycine contributes to inflammation by promoting toll-like receptor 2 to release increased levels of TNF-α (Nichols et al., 2020), and upregulating bone resorption stimulators prostaglandin E2 and cyclooxygenases in periodontitis (Rausch-Fan et al., 2005). Further work to investigate the role of proline in immune modulation due to glycine utilization is necessary.

Previous studies outside of the oral cavity have shown differing effects of proline on biofilm growth and formation. In *E. coli* (Goh et al., 2013) proline has been shown to induce growth of biofilms at high concentrations (0.4% of growth medium) and increase swimming and twitching motility (necessary for biofilm formation) by up to 100%. In *S. aureus*, one study showed that D-proline has an inhibitory effect on biofilm formation—specifically proliferation of the biofilm and not initial attachment (Hochbaum et al., 2011). In *Pseudomonas aeruginosa*, short proline-rich peptides have been shown to inhibit cyclic-di-guanosine monophosphate, which is crucial for biofilm formation in this organism (Foletti et al., 2018).

The mouth is generally described as a nutrient-limited environment in between meals, as the constant flow and buffering capacity of saliva removes dietary energy sources that can be utilized by bacteria. Without a readily available and easy to metabolize energy source, such as sucrose or glucose, oral bacteria must metabolize other nutrient sources, such as high molecular weight glycoproteins and other salivary proteins, to provide carbon and nitrogen. Although a recent large study divided oral metabolism into proteolytic and saccharolytic (Zaura et al., 2017), proteolysis is often associated with pathogenic bacteria such as *P. gingivalis* in gingivitis. However, pathogenic and non-pathogenic bacteria that are associated with the breakdown of glucose and sucrose have also been described as proteolytic. In one study, *S. mutans* strains were shown to have increased acid tolerance response in low carbohydrate but amino acid rich growth-medium via increased proteolysis and amino acid metabolism (Svensäter et al., 2001). A consortium of *Streptococcus mitis, Streptococcus bovis, Streptococcus gordonii* and *Actinomyces neaslundii* have been shown to preferentially proteolytically degrade the salivary protein mucin 5b, likely by combined protease and glycosidase activity (Wickstörm et al., 2009). *S. gordonii* has been shown to produce an extracellular protease, in response to growth media that are low in amino acids (Juarez and Stinson, 1999). *Streptococcus sanguis* biofilms have also been shown to degrade salivary proteins (De Jong and Van Der Hoeven, 1987) via glycosidase, protease and esterase activity. Based on these studies, the increased loss of salivary proteins seen in day 4 glucose 10 mM supplemented biofilms might not be that unexpected. As with our previous findings (Cleaver et al., 2019), we found unsupplemented *in vitro* bacterial biofilms inoculated from healthy stimulated whole mouth saliva and grown in saliva as the sole nutrient source will readily degrade salivary proteins. What we have shown here for the first time by using labeled substrates is that these biofilms, derived from a saliva inoculum, will metabolize amino acids into metabolites typically found in saliva. A potential feedback mechanism is also revealed here whereby, when supplemented with proline or arginine, the biofilms significantly reduced protein degradation in immature biofilms. It has previously been shown that when bacteria degrade salivary proteins, particularly in periodontitis, the levels of 5-aminopentanoate increase (Syrjänen et al., 1987). In this study, the levels of 5-aminopentanoate being produced by proline supplemented biofilms were similar to the unsupplemented control, yet the degradation of salivary proteins was reduced. The 1D and 2D NMR spectral analysis indicated that butyrate was also produced, in statistically greater concentrations, in proline supplemented biofilms—therefore, 5-aminopentanoate should be used with caution as an indicator of salivary protein degradation. Despite the carbohydrate availability in glucose supplemented biofilms, which might suggest decreased degradation of salivary proteins by glycosidases (Inui et al., 2015), there was only significant inhibition of protein degradation at day 7 of biofilms supplemented with 50 mM glucose.

The beneficial effects of arginine to oral health are linked to increased pH of plaque biofilms through the bacterial arginine deiminase pathway (Lu, 2006). As arginine is a basic amino acid and negatively charged at physiological pH it was more alkali at day 0 than unsupplemented biofilms. As it became metabolized by day 4 the pH returned toward neutral by day 7. The NMR data suggested arginine supplemented biofilms are utilizing all of the available arginine throughout the experiment (data not shown). There are two different pathways present in certain strains of bacteria where arginine is catabolised into putrescine (Lu, 2006): (1) L-arginine→L-ornithine (production of urea/ammonia)→putrescine, and (2) L-arginine→agmatine (production of CO_2_)→putrescine. In this experiment, the pH became more acidic as biofilms supplemented with arginine matured, whilst the putrescine produced by these biofilms remains significantly increased, and this could possibly be due to bacteria utilizing the second mentioned pathway and producing more CO_2_, or via the first pathway with the subsequent utilization of urea by urease producing bacteria. A limitation of this study is that NMR does not identify ammonia, or intracellular metabolites. This would have to be confirmed through ammonia identification or urease activity (colorimetric assay).

Unsurprisingly, the supplementation of biofilms with increased glucose concentrations significantly decreased the pH of the biofilm (Takahashi and Yamada, 1999; Senneby et al., 2017), presumably due to the production of lactic acid/lactate. These results align with a recent study which showed that biofilms supplemented with glucose can survive up to 12 days of incubation at pH 4.1 (Du et al., 2020). In contrast to arginine and glucose which both altered the pH of the media, proline maintained the neutral pH despite the production of acidic metabolites such as butyrate and acetate. One limitation of this study is that pH is analyzed by measuring the spent saliva from biofilm growth. The use of a ratiometric pH probe, C-SNARF-4 (Schlafer et al., 2015), could be employed in future work to measure the pH of biofilms horizontally through the complex 3D structure of the biofilm to comprehend whether proline maintains biofilm neutrality within the biofilm architecture.

Takahashi et al. succinctly describe the emerging trend of the metabolomic study of oral biofilms and microbiomes as less of “who are they?” and more of “what are they doing?” which summarizes the aims that we have succeeded in investigating in this study (Takahashi et al., 2012; Takahashi, 2015). One limitation of our study is the absence of 16S rRNA gene analysis to understand whether proline is promoting the growth of non-pathogenic oral species. This will be combined in a separate study with metatranscriptomics to examine which genes are up/downregulated when biofilms are supplemented with proline. Another limitation of this study is the large quantities of saliva required for each experiment. This has limited the comparisons, but an interesting next step might be to examine the possible effects of proline on glucose supplemented biofilms for a possible anti-caries effect.

We have shown in this study that proline is readily metabolized by multispecies biofilms affecting growth and salivary protein degradation. Proline partially fits the most up-to-date definition of a prebiotic, and further work will be performed to gain a deeper understanding of its mechanism of action and how it benefits the host. These results contrast previous findings outside of the oral cavity which have particularly focused on the elimination or inhibition of medical device biofilms using proline. Biofilms in the oral cavity can never be completely eliminated, although regular brushing and removal of plaque buildup aids in the control of mature biofilms *in situ*. Many studies have attempted to completely eradicate the bacteria present in the mouth through the use of antimicrobials, photodynamic therapy and ultrasonication. However, reservoirs of oral bacteria at sites such as the tongue (Takahashi, 2005; Matsui et al., 2014) will always repopulate the mouth with bacteria that can then reattach to other dental surfaces. With this in mind, perhaps it is pertinent to stop attempting to remove oral biofilms completely, and to instead to control their activity through the use of prebiotics.

## Data Availability Statement

The datasets generated for this study can be found in online repositories. The names of the repository/repositories and accession number(s) can be found below: The mass spectrometry and NMR data have been deposited in the Metabolomics Workbench (http://www.metabolomicsworkbench.org/) under project ID PR000984. The data can be accessed directly via its project, doi: 10.21228/M8H68N.

## Ethics Statement

The studies involving human participants were reviewed and approved by King's College London Research and Ethics Committee, reference HR-17/18-6116. The patients/participants provided their written informed consent to participate in this study.

## Author Contributions

LC contributed to the design of the study, undertook experiments, analyzed the data, and wrote the manuscript. RM and GC contributed to the design of the research, analysis of the data, supervision of the project, and the final draft of the manuscript. All authors contributed to the article and approved the submitted version.

## Conflict of Interest

The authors declare that the research was conducted in the absence of any commercial or financial relationships that could be construed as a potential conflict of interest.

## Supplementary Material

Supplementary Figure 1 Confocal laser scanning microscopy images of unsupplemented and supplemented biofilms on hydroxyapatite discs stained with SYTO-9 and propidium iodide (x63 magnification, live bacteria are green, dead bacteria are red).

Supplementary Figure 2 Coomassie-stained SDS-PAGE gel for unsupplemented (positive control) biofilms and incubated sterile saliva (negative control) at days 4 and 7, with the sterile saliva standard in wells one to three (*n* = 3).
