# Supplementary figures and images for: Evidence for Proline Utilization by Oral Bacterial Biofilms Grown in Saliva
Source: Front Microbiol. 2021 Jan 20;11:619968. doi: 10.3389/fmicb.2020.619968 (PMC7855038; doi:10.3389/fmicb.2020.619968)

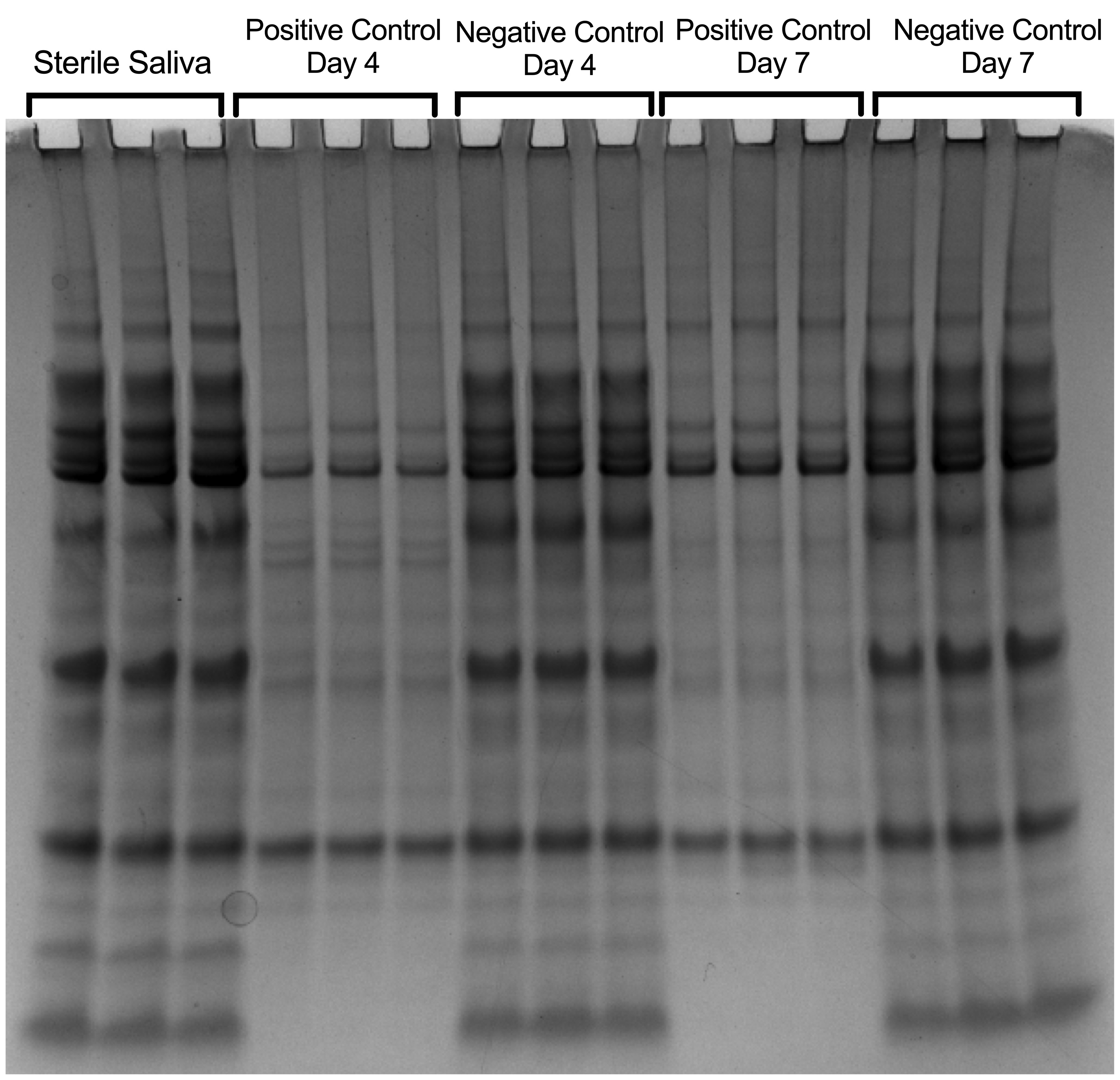

Supplement: Supplementary Figure 2 — Coomassie-stained SDS-PAGE gel for unsupplemented (positive control) biofilms and incubated sterile saliva (negative control) at days 4 and 7, with the sterile saliva standard in wells one to three (n = 3). [file Image_2.JPEG]
